# Torsion of the wandering spleen as an abdominal emergency: a case report

**DOI:** 10.1186/s12893-021-01289-x

**Published:** 2021-06-09

**Authors:** Matiullah Masroor, Mohammad Arif Sarwari

**Affiliations:** 1 Department of General Surgery, Amiri Medical Complex, Qargha Road, Afshar, Kabul, Afghanistan; 2 Department of Cardiovascular Surgery, The Second Xiangya Hospital of Central South University, 139 Renmin Middle Rd, Changsha, 410011 China

**Keywords:** Wandering spleen, Pelvic spleen, Splenectomy, Acute abdomen, Case report

## Abstract

**Background:**

Wandering spleen is a rare clinical entity with a less than 0.2% reporting incidence rate. In this case, the spleen is present abnormally in the abdominal or pelvic cavity instead of its normal anatomical location. The aetiology is either congenital or acquired. The condition is caused by the absence or maldevelopment of the spleen's suspensory ligaments, which holds the spleen static in the left hypochondrium.

**Case presentation:**

A 27-year-old female patient presented to the emergency department with complaints of abdominal pain, fever, nausea, vomiting, and constipation for three days. A palpable movable mass was found during the physical examination, and torsion of the wandering spleen’s pedicle was confirmed by CT scan. Open splenectomy was performed, and the patient was recovered uneventfully.

**Conclusion:**

Even though ectopic spleen is a rare disease, clinicians should be aware of its incidence. Early diagnosis in the case of an acute abdomen is vital for the preservation of the spleen. Patients presented with acute abdomen and absence of splenic shadow under left hemidiaphragm should be suspected, and further radiological investigation will confirm the diagnosis. Surgery is the gold standard for wandering spleen with either splenopexy or splenectomy, depending on the spleen's condition during surgery.

## Background

wandering spleen is a rare clinical entity where the spleen is found abnormally somewhere else in the abdominal cavity or pelvic cavity instead of its normal anatomical position in the left hypochondrium. The disease does not have any genetic background but can either be congenital or acquired [1]. The aetiology is the absence, malformation or hyperlaxity of one or more splenic ligaments which holds the spleen static in the left hypochondrium [2]. It can present with various clinical pictures ranging from asymptomatic to acute abdomen, which need immediate surgical intervention. In asymptomatic patients, the diagnosis may be incidental when the patient needs a medical checkup for any other reason. In symptomatic cases, it may diagnose with complications like torsion of the spleen, which can lead to splenomegaly, infarction, splenic rupture, hemoperitoneum, and peritonitis [3, 4].

It can be diagnosed easily and can be sometimes challenging too, especially in children, due to ambiguous presentation. Because the symptoms are very nonspecific radiological investigations are of great help. Plain x-ray, doppler ultrasonography, computerized tomography(CT), magnetic resonance imaging(MRI), scintigraphy, and splenic angiogram are very helpful in diagnosis [5–7]. Timely diagnosis and interventions are of vital importance to save the spleen. Open or laparoscopic surgery is the gold standard of treatment for ectopic spleen. The choice is splenopexy when there is no infarction, splenomegaly or hypersplenism, and splenectomy when any of these complications are present [3].

Here we present a 27-year-old female patient referred to the emergency room of Amiri Medical Complex, Kabul, Afghanistan, from a rural area of the country.

## Case presentation

A 27-year-old, multiparous, female, poor patient with a history of umbilical herniorrhaphy eight years ago, and otherwise healthy, presented to our emergency department with acute onset abdominal pain, nausea, vomiting, constipation, on and off fever, and urinary retention from last three days. The pain was relieving with analgesics but exaggerated with physical activity. She said, she experienced the same episode of pain two years ago but was diagnosed as gastritis on endoscopy, for which she was treated. The sonologist at that time suggested her to seek a surgeon's opinion, but she ignored it. She had no previous records with her at the time of admission. Drug and family history was unremarkable.

The vital signs were BP 110/70 mmHg, RR 22/m, PR 70 b/m, Temp 37.5 °C, and SpO_2_ 97%. On physical examination, the abdomen was mildly distended with a midline surgical scar and hypoactive bowel sounds on auscultation. A mobile palpable mass with a smooth surface in the right lower abdomen extending into the pelvis was observed, which was painful on movement. No signs of peritonitis were observed. Blood investigation results were TLC 11,000/mm^3^ (normal 4000–11,000), neutrophil 83% (normal 40–75), Hb 12.8 g/dl (normal 11.5–16.5), INR 1.18, blood group A + , anti HCV Ab + , HbsAg, and HIV were negative.

**Fig.1:**
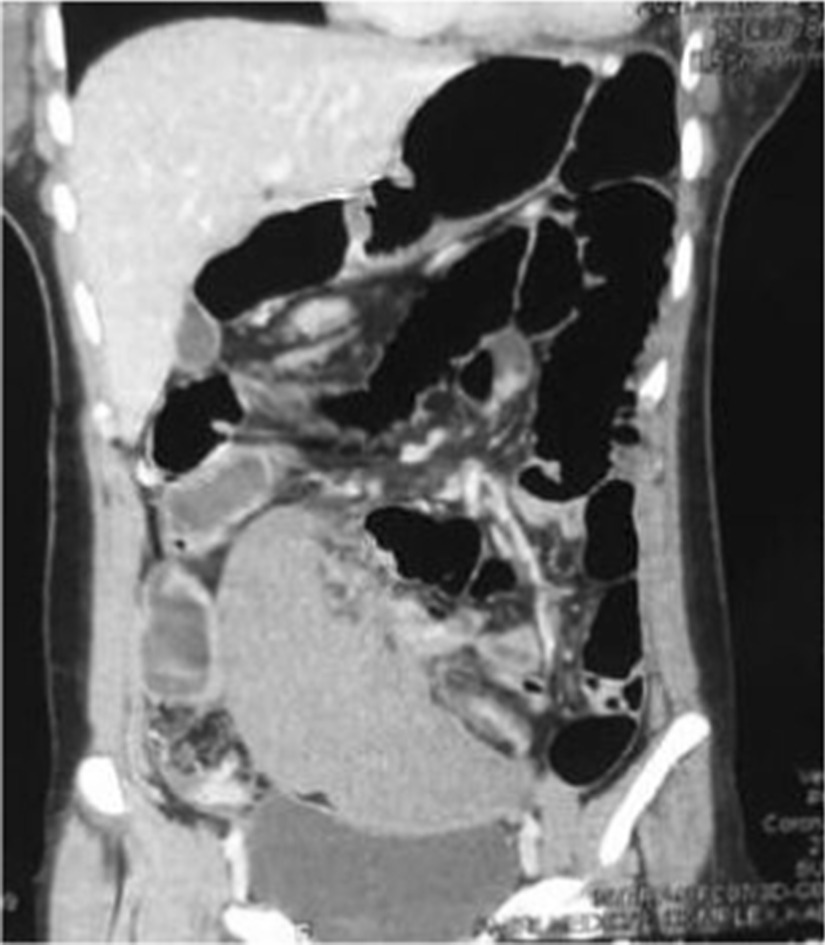
A coronal CT section showing empty left hemidiaphragm distended small and large bowel, misplaced non enhanced mildly enlarge spleen in the right lower abdomen extending into the pelvis, resting above the urinary bladder and bright capsule (rim sign) of the surface adjacent to the bladder

**Fig.2:**
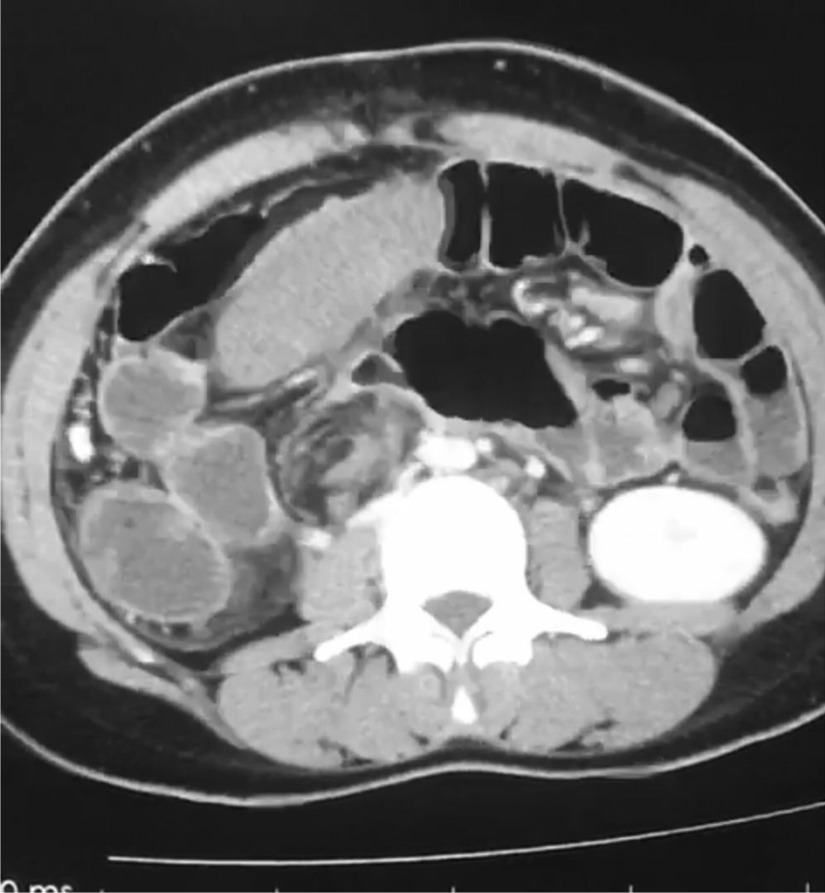
An axial section of the CT scan showing a "whirl sign" (pathognomonic for pedicle torsion) of the splenic pedicle

Ultrasound result was not diagnostically significant, so an abdominal CT scan with and without contrast was advised, which showed an empty splenic fossa and mildly enlarged ectopic spleen measuring 15.5 cm in length, present in the right lower abdomen extending into pelvis resting on the bladder fundus. No enhancement of the spleen was noted after iv contrast. The bright capsule of the spleen was observed, indicating collateral flow (rim sign). Regional ileus of the small and large bowel was also seen, as shown in Fig. 1. A whirl sign indicating torsion of the pedicle of the spleen was observed on the axial section, as shown in Fig. 2. Other advanced technologies for investigations such as scintigraphy and splenic angiography were not available, and the patient could not afford them as well.

**Fig.3:**
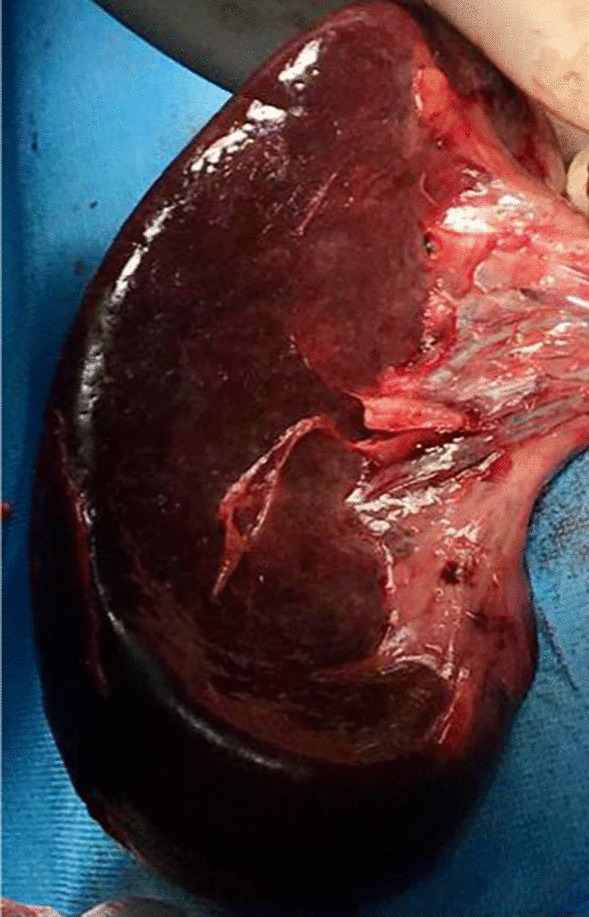
Intraoperative photograph of the mildly enlarged, congested and ischemic spleen after splenectomy

**Fig.4:**
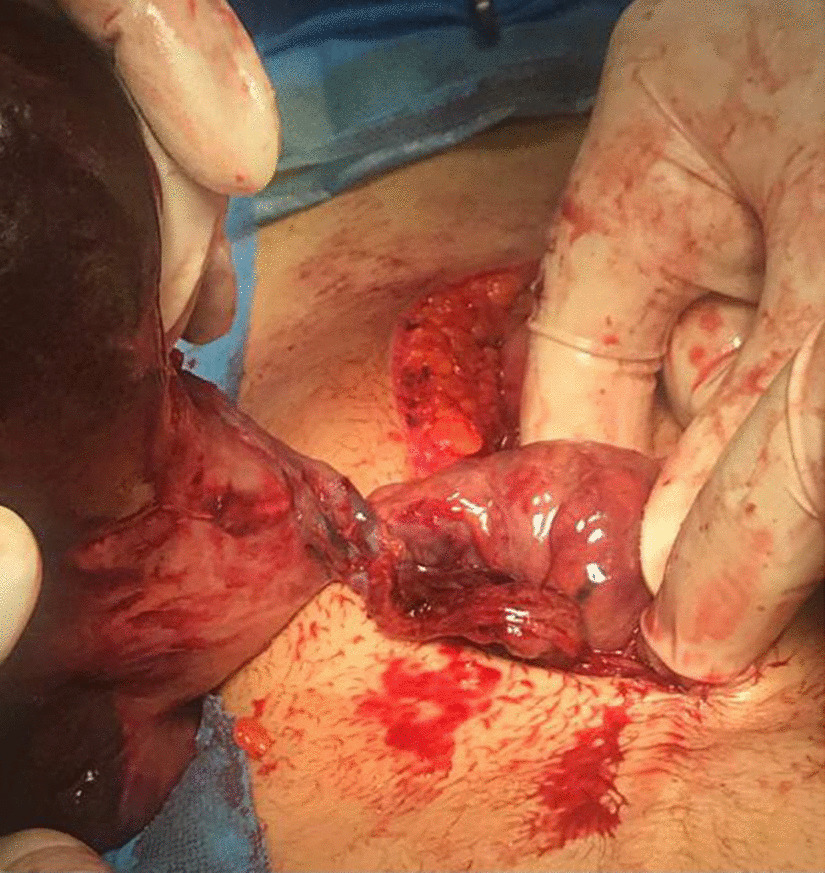
Torsion of the prolonged splenic pedicle just before splenectomy

**Fig.5:**
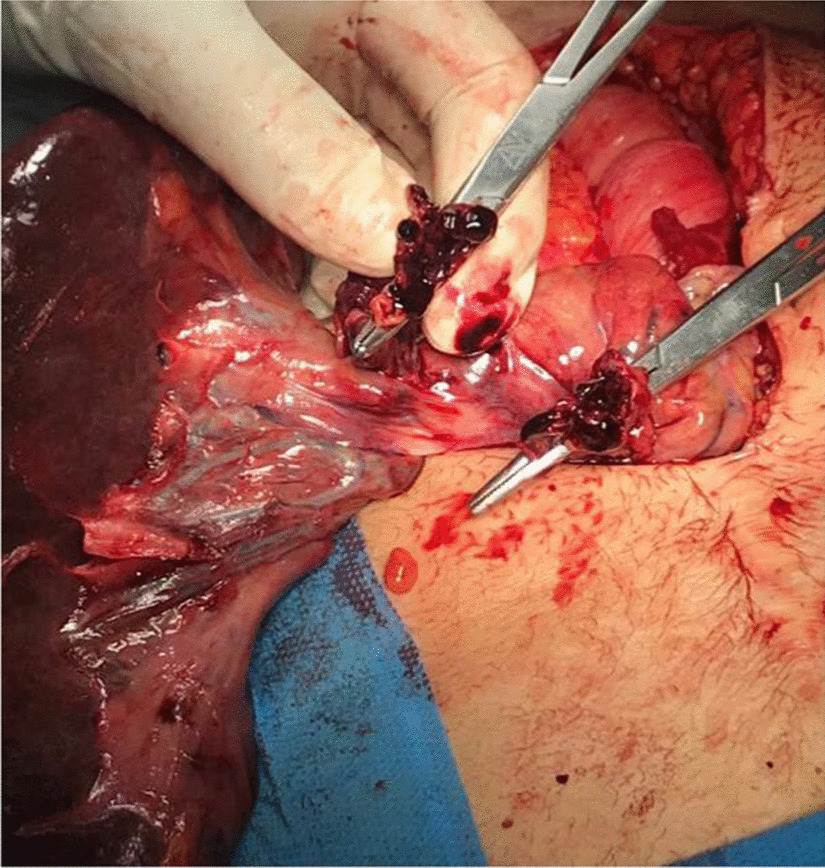
Thrombosis of the splenic vessels, right after cutting the pedicle of the spleen during splenectomy

Laparoscopic surgery was not feasible because of lack of technology and expertise, so laparotomy was performed, and mildly enlarged congested and ischemic spleen with 180^0^ twisted pedicle was found, as shown in Figs. 3 and 4. After detorsion of the spleen and waiting for some time, no colour change was observed. The spleen was not viable, and a splenectomy was performed, which showed thrombosed splenic vessels, as shown in Fig. 5.

The patient recovered well without any complication and was discharged on 3rd post-operative day. She was followed up for a month without any complaints.

## Discussion and conclusion

Wandering spleen, which is also called floating spleen, ectopic spleen, ptotic spleen, or splenoptosis, can wander anywhere in the abdominal or pelvic cavity attached only by its vascular pedicle with no attachment to the surrounding structures. The long pedicle makes it liable to torsion on itself and can cause complications. The degree of torsion, according to literature, ranges from 90° to 2160^o^ [8]. The causes of the ectopic spleen are either congenital or acquired. The congenital causes are the developmental anomalies of dorsal mesogastrium, which does not fuse with posterior peritoneum during the fifth and sixth week of fetal development leading to absence or abnormal development of one or more suspensory splenic ligaments (gastrosplenic, phrenocolic, and splenorenal). Acquired causes which are believed to cause laxity of these ligaments include connective tissue disorders, multiparity, hormonal changes, splenomegaly, trauma, and abdominal wall weakness [1–5].

The concept of wandering spleen was first described by Van Horne in 1667 [9]. One of the first documented cases of wandering spleen in a child was published by Jozef Dietl, a polish physician, in 1854 [10]. Children make one-third of all the cases of wandering spleen, and under the age of ten, there is no gender difference in terms of occurrence, but in adults, it is more common in women than men. Abell Irvin, in 1933, by analysis of 93 wandering spleen cases with pedicle torsion, concluded that 88 cases occurred in women, most of them were of childbearing age, ranging from 21 to 40 years, and the mortality rate was 17.6% in patients with splenectomy [11]. The higher incidence in women of childbearing age is associated with hormonal changes, multiple pregnancies, and abdominal wall weakness [5]. It is a rare entity with a reported incidence rate of less than 0.2% and accounts for 0.002% (two per thousand) splenectomies [12]. Till now, less than 600 cases of wandering spleen have been reported in literature ranging from 3 months to 82 years.

The presentation is very nonspecific. Asymptomatic cases may be left undiagnosed or diagnosed incidentally. Symptomatic patients may have different clinical signs and symptoms as reported in the literature, e.g. acute, intermittent, or chronic pain because of torsion and detorsion of the splenic pedicle, vomiting, abdominal distention, constipation, palpable, movable mass in the abdomen or pelvic same like in our case or other signs and symptoms of an acute abdomen may present [2–4, 13–15]. The symptoms may be related to the kind of complication patients present with; for example, if a patient presents with a wandering spleen leading to intestinal obstruction, the patient may have pain, abdominal distention, vomiting, and constipation. If it presents with torsion of the pedicle leading to infarction or splenic rupture, the patient may have signs and symptoms of peritonitis and maybe in shock because of hemoperitoneum. Association of wandering spleen with gastric volvulus, pancreatic volvulus, portal hypertension, mesenteric varices, and horseshoe kidney has been reported in the literature [5, 8, 12].

All the signs and symptoms presented in our patient were not different from the literature. While we were investigating the cause of abdominal pain and found the palpable, smooth surface, mobile mass on physical examination, it was considered the definitive cause for abdominal pain. Keeping her age and gender in mind, the first impression was the torsion of ovarian cyst. But after an ultrasound showed a complex mass in the right lower abdomen and absence of splenic shadow from splenic fossa on x-ray, the diagnosis of wandering spleen was more likely, which was finally diagnosed on CT scan and confirmed during operation. The acute pain was because of torsion of the splenic pedicle leading to ischemia of the spleen. Abdominal distention, nausea, vomiting, and constipation with hypoactive bowel sounds on auscultation were possibly because of paralytic ileus. The patient was not having the signs of peritonitis because the spleen was not infarcted and ruptured. But peritonitis can occur as a complication of torsion. Almost all the symptoms of our case were in compliance with the literature and have already been explained above.

The diagnosis can be made by doing a proper physical examination, ultrasonography, CT scan, MRI, scintigraphy, and angiography, etc. The absence of the spleen from its normal anatomical position and the presence of soft tissue splenic shape mass in the abdomen or pelvis on a CT scan is suggestive of a wandering spleen. In addition, a "whirl sign" with no or partial enhancement of the splenic shape mass on iv contrast-enhanced CT scan in the patient presented with the acute abdomen is strongly suggestive for pedicle torsion of the wandering spleen, as also seen in our case [7]. Because it is a rare entity and radiologist can misdiagnose it if he/she does not consider it and pay much attention to it. Meanwhile, surgeons' experience plays a vital role in the proper diagnosis and treatment of the patient.

Timely diagnosis and interventions are vital for spleen salvage and avoiding life-threatening complications. Thrombocytopenia is another rare complication usually related to torsion of elongated splenic pedicle [6].

The gold standard treatment for wandering spleen is surgery. Conservative management of asymptomatic wandering spleen is associated with a 65% complication rate [3]. The two options available for surgeons are splenopexy and splenectomy, depending on the conditions of the wandering spleen. The first documented splenectomy was performed by Martin BA in 1877 [16], and in 1895, Ludwick Rydygier performed the first splenopexy to fix the wandering spleen to the peritoneum [17]. The best and easiest procedure for splenopexy is Bardenheuer's procedure, where the spleen lies in the retroperitoneal pouch with the body hanging from the tenth rib and the pedicle attached to the peritoneal incision [6]. After the advancement in laparoscopic abdominal surgeries, the new gold standard is laparoscopic splenopexy, when the wandering spleen is of normal size, not infarcted, and has no signs of hypersplenism [14]. The sandwich technique where two meshes are used to sandwich the spleen is described in the literature for laparoscopic splenopexy [18].

When the spleen is enlarged, infarcted, ruptured, or there are signs of hypersplenism, the choice of treatment is splenectomy, where the spleen should be totally removed either laparoscopically or through laparotomy [2–4].

Wandering spleen is a comparatively rare clinical entity, which can present with various signs and symptoms. Emergency room physicians and surgeons should be aware of this entity, especially when the patient presents with the acute abdomen. The absence of the spleen from its normal anatomical location and the presence of soft tissue splenic shape mass anywhere else in the abdomen or pelvis is the likely diagnosis of a wandering spleen. Whirl sign and partial or no enhancement of splenic shape mass on contrast-enhanced CT scan is the likely diagnosis for pedicle torsion of the ectopic spleen in patients presenting with acute abdomen. Timely diagnosis and interventions are crucial for spleen preservation and avoiding life-threatening complications. Surgery for splenopexy or splenectomy is the treatment of choice depending on the condition of the spleen.

## Data Availability

The datasets used during the current study are available from the corresponding author on reasonable request.

## Abbreviations

CT: Computerized tomography
MRI: Magnetic resonance imaging
USG: Ultrasonography
